# Automated registration of dynamic contrast enhanced DCE-MRI cardiac perfusion achieves comparable diagnostic accuracy to manual motion correction: a CE-MARC sub-study

**DOI:** 10.1186/1532-429X-16-S1-P344

**Published:** 2014-01-16

**Authors:** Constantine Zakkaroff, Aleksandra Radjenovic, John D Biglands, Sven Plein, John P Greenwood, Derek R Magee

**Affiliations:** 1 Division of Medical Physics, University of Leeds, Leeds, UK; 2 School of Computing, University of Leeds, Leeds, UK; 3 MCRC & LIGHT, University of Leeds, Leeds, UK; 4 Institute of Cardiovascular and Medical Sciences, BHF Glasgow Cardiovascular Centre, University of Glasgow, Glasgow, UK

## Background

The human interaction required for manual motion correction/contouring of cardiac perfusion series remains a significant obstacle to quantitative perfusion gaining a wider acceptance in clinical practice. The use of image registration for motion correction in perfusion data offers a considerable time saving. Numerous registration methods have been proposed, with evaluation limited to the image registration accuracy. However, the important clinical question is how do these methods affect diagnosis? The aim of this study is to evaluate perfusion series registration in terms of its affect on the diagnostic accuracy of myocardial ischaemia.

## Methods

**Figure 1:**
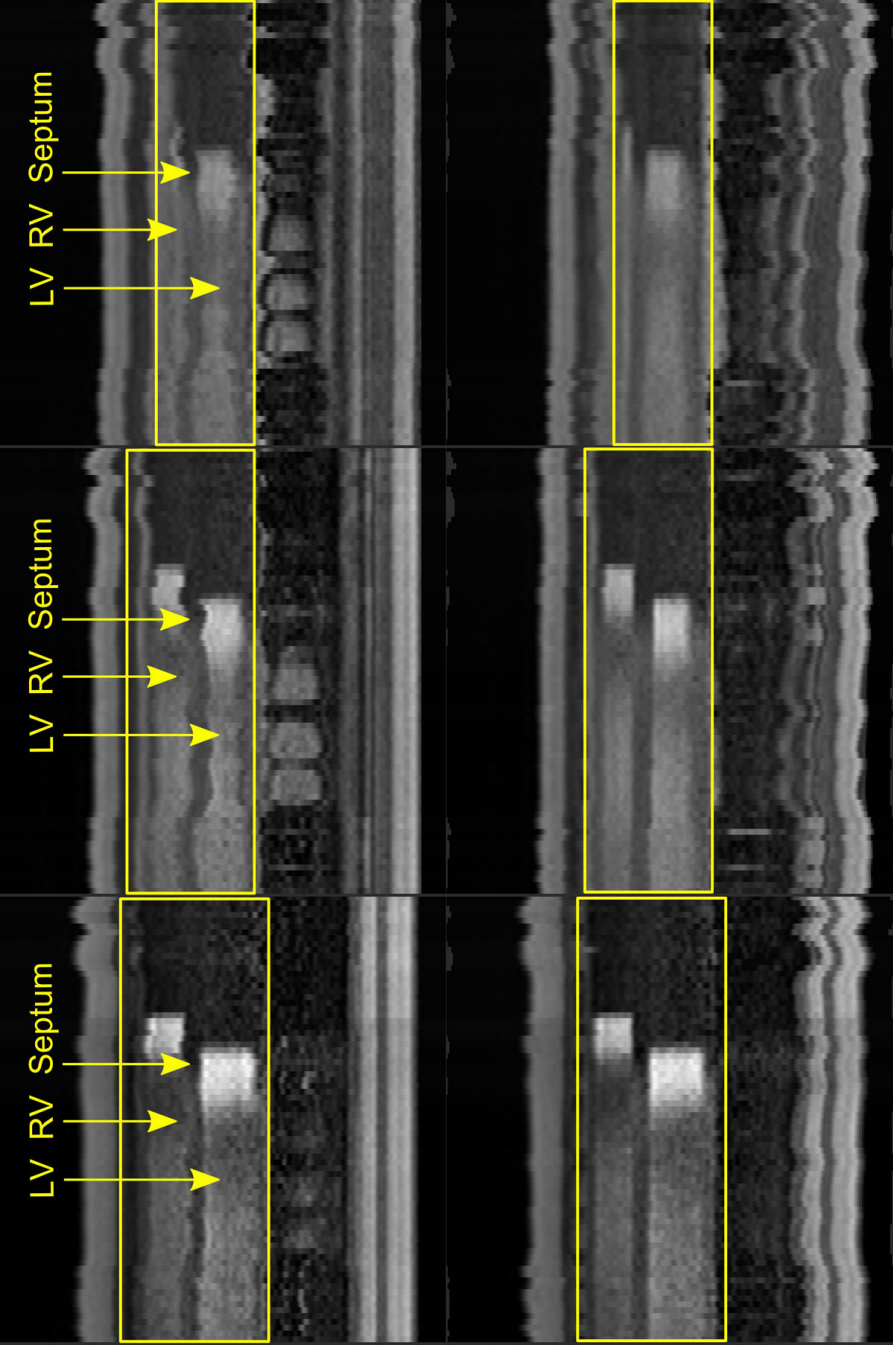
**Reformatted perfusion series (apical, medial and basal slices) before (left) and after motion correction (right); the regions of interest (yellow rectangles) include the right ventricle (RV), left ventricle (LV) and interventricular septum; the magnitude of motion in the series before correction can be observed through the shifting position of the septum**.

This was a retrospective sub-study using data from the CE-MARC trial (Greenwood et al., Lancet, 2012). A 50-patient sample was selected such that the distribution of risk factors and disease status within the sample was representative of the full CE-MARC cohort. Image registration was performed with the mutual information image similarity metric; all images in the basal location of the series were registered with translation displacement to the maximum contrast image of the series at the basal slice. The recovered correction transforms were propagated to the medial and apical slices of the series (Figure 1). Contours describing the myocardium and a region within the left blood pool were drawn on all slices and dynamic frames, manually correcting for motion. Signal vs. time curves were generated for the manual and automatic motion-correction methods. The resulting time varying signal curves were used to generate quantitative myocardial blood flow (MBF) estimates using Fermi constrained deconvolution. Myocardial perfusion reserve (MPR) indices were calculated from the ratio of stress to rest MBF estimates. The presence of myocardial ischaemia was assessed using the consensus diagnosis of invasive, quantitative X-ray angiography and myocardial Single Photon Computed Tomography (SPECT) imaging. This provided a unique gold-standard combining independent anatomical and functional diagnostic measures. Receiver Operator Characteristic (ROC) curves were generated using the MPR indices. A DeLong, DeLong, Clarke-Pearson comparison was used to test for statistically significant differences in the area under the curve (AUC) values between the registered and manually corrected ROC curves.

## Results

**Figure 2:**
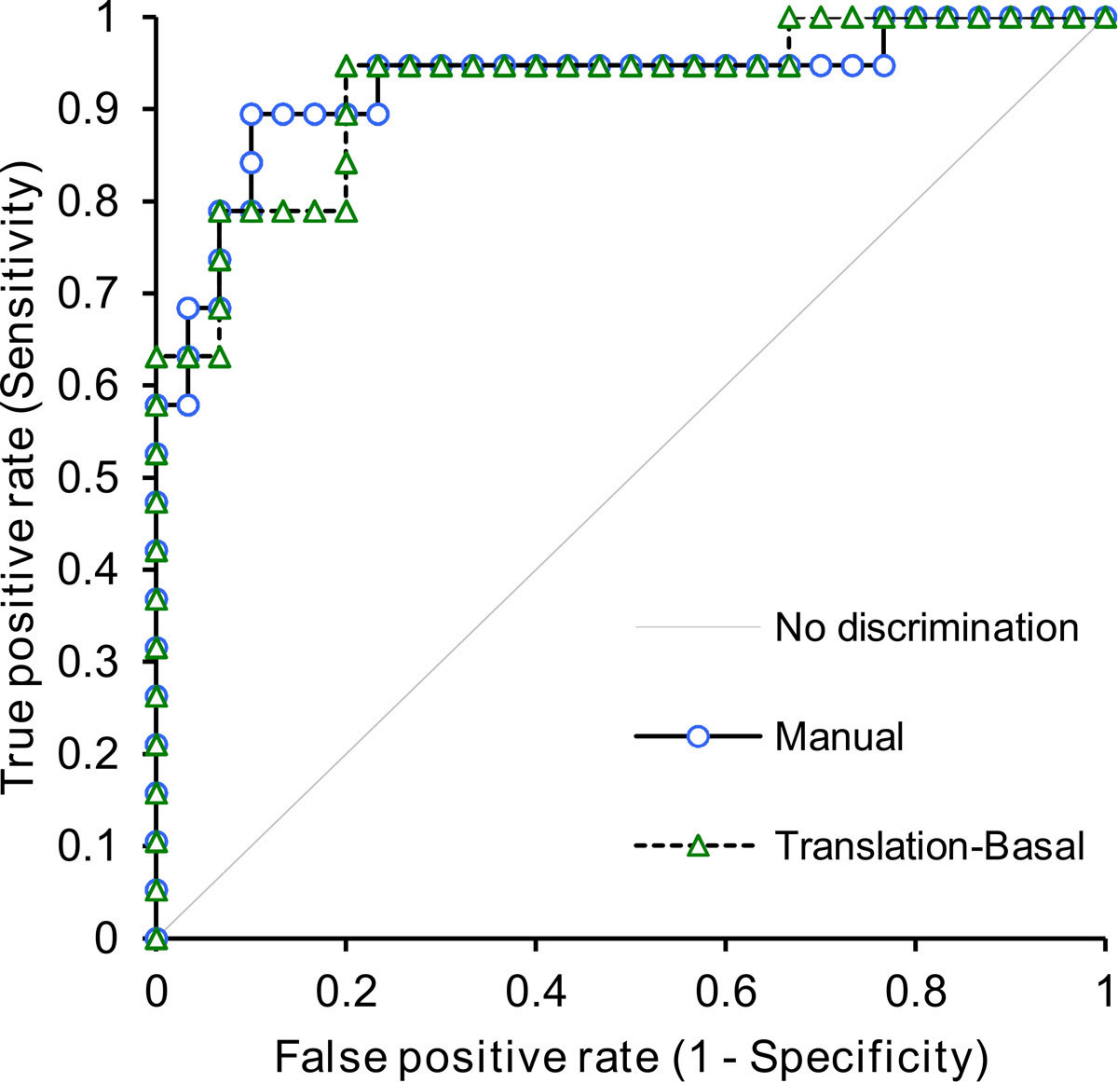
**ROC curves generated for manual and automatic motion correction**.

There were no significant differences in diagnostic accuracy between the manual and automatic motion-corrected datasets (p = 0.88). The AUCs for manual motion correction and automatic motion correction were 0.93 and 0.92 respectively (Figure 2).

## Conclusions

We have shown that automated motion correction provides diagnostic accuracy equivalent to the common protocol of manual motion correction. Automated motion correction offers a significant time reduction in the human interaction required for delineation of contours for quantitative perfusion analysis, and therefore opens the way for a more widespread use of this technique in research and clinical practice.

## Funding

This work was funded by the Top Achiever Doctoral scholarship awarded by the Tertiary Education Commission of New Zealand.

